# Occurrence of *Babesia* and *Anaplasma* in ruminants from the Catimbau National Park, Semiarid Region of Northeast Brazil

**DOI:** 10.1590/S1984-29612024062.

**Published:** 2024-10-07

**Authors:** Liliane Moreira Donato Moura, Ila Ferreira Farias, João Claudio Bezerra de Sá, Dênisson da Silva e Souza, Paula Talita Torres Santos, Carla Roberta Freschi, Jaqueline Bianque de Oliveira, Jonas Moraes-Filho, Rosangela Zacarias Machado, Sergio Santos de Azevedo, Mauricio Claudio Horta

**Affiliations:** 1 Laboratório de Doenças Parasitárias, Universidade Federal do Vale do São Fracisco - Univasf, Campus Ciências Agrárias, Petrolina, PE, Brasil; 2 Laboratório de Parasitologia, Departamento de Biologia, Universidade Federal Rural de Pernambuco - UFRPE, Recife, PE, Brasil; 3 Imunodot Desenvolvimento, Indústria e Comércio de Imunógenos e Produtos Veterinários Ltda, Jaboticabal, SP, Brasil; 4 Programa de pós-graduação em Saúde Única, Universidade Santo Amaro - Unisa, São Paulo, SP, Brasil; 5 Departamento de Patologia Veterinária, Universidade Estadual Paulista - UNESP, São Paulo, SP, Brasil; 6 Unidade Acadêmica de Medicina Veterinária, Universidade Federal de Campina Grande - UFCG, Patos, PB, Brasil

**Keywords:** Conservation unit, epidemiology, hemoparasites, ruminants, ticks, Unidade de conservação, epidemiologia, hemoparasitas, ruminantes, carrapatos

## Abstract

Babesiosis and Anaplasmosis are diseases associated with economic losses; ticks and blood-sucking flies are important zoonotic vectors and reservoirs. This study aimed to investigate the presence of anti-*Babesia* spp*.* and anti-*Anaplasma marginale* antibodies using enzyme-linked immunosorbent assay (ELISA), in ruminants at the Catimbau National Park. Blood samples were collected from 119 sheep, 119 goats, and 47 cattle. *Rhipicephalus microplus* ticks were collected from cattle. ELISA showed seropositivity of 34% (16/47), 20.3% (24/119), and 16% (19/119) for anti-*Babesia bovis*; 34% (16/47), 15.2% (18/119), and 9% (7/119) for anti-*Babesia bigemina*; and 34% (16/47), 35.6% (42/119), and 17% (20/119) for anti-*A. marginale* antibodies in cattle, goats, and sheep, respectively. The information collected using an epidemiological questionnaire showed that mostly are breed in a semi-intensive system, with access to Caatinga vegetation. The circulation of *B. bovis, B. bigemina,* and *A. marginale* was confirmed. Thus, based on the prevalence, this suggests this is an enzootic instability area and is prone to outbreaks.

## Resumo

A Babesiose e a Anaplasmose são doenças associadas a grandes perdas econômicas, sendo carrapatos e moscas sugadoras de sangue importantes vetores e reservatórios zoonóticos dessas enfermidades. Este estudo teve como objetivo investigar a circulação de anticorpos anti-*Babesia* spp. e anti-*Anaplasma marginale* usando Ensaio de Imunoabsorção Enzimática (ELISA), e *Babesia* spp. em ruminantes no Parque Nacional do Catimbau. Foram coletados ectoparasitos e amostras de sangue de 119 ovinos, 119 caprinos e 47 bovinos. Carrapatos *Rhipicephalus microplus* foram coletados em bovinos. O teste ELISA mostrou soropositividade de 34% (16/47), 20,3% (24/119) e 16% (19/119) para anti-*Babesia bovis*; 34% (16/47), 15,2% (18/119) e 9% (7/119) para anti-*Babesia bigemina*; e 34% (16/47), 35,6% (42/119) e 17% (20/119) para anti-*A. marginale* em bovinos, caprinos e ovinos, respectivamente. As informações coletadas por meio de questionário epidemiológico mostraram a maioria com sistema semi-intensivo, com acesso à vegetação de Caatinga. A circulação de *B. bovis, B. bigemina* e *A. marginale* foi confirmada. Assim, com base na prevalência, essa área foi sugerida como de instabilidade enzoótica e propensa a surtos.

## Introduction

The Catimbau National Park (CNP) is part of the municipalities of Buíque, Tupanatinga, and Ibimirim in the state of Pernambuco, a semiarid region of Brazil. It's known to have endemic and rare species of plants and animals in the morphoclimatic domain of the Caatinga biome (Rodal et al., 1998). Although the CNP is a conservation unit (CU), it is inhabited by smal farmers (Azevedo & Bernard, 2015), indigenous groups, and livestock; it also features agricultural production and ecotourism (Azevedo & Bernard, 2015; Fonseca & Freire, 2017).

Livestock is prone to the emergence of diverse ectoparasites that can host different pathogens between animals and humans. Bovine babesiosis is caused by *Babesia bovis* and *Babesia bigemina* in ruminants, and the tick *Rhiphicephalus microplus* is its main vector in Latin America (Estrada-Peña et al., 2006; Ríos-Tobón et al., 2014). Another disease that can affect livestock is Anaplasmosis, caused by blood parasites belonging Anaplasmataceae family (order Rickettsiales), such as *Anaplasma marginale* (Ristic, 1981).

Cattle tick fever (CTF) syndrome is an infection or co-infection involving these three agents: *B. bovis, B. bigemina* and *A. marginale* (Guglielmone, 1995; Costa et al., 2013) that causes morbidity, mortality, and economic losses owing to its treatment and prevention (Martins et al., 2020). This syndrome is more common in tropical and subtropical areas, especially in cattle, buffalo, and small ruminants (Nayel et al., 2012; Sivakumar et al., 2013). Parasites in livestock in Brazil can lead to an annual loss of approximately US$ 13.96 billion (Grisi et al., 2014).

To the best of author’s knowledge, there are no reports of *B. bigemina* and *B. bovis* in small ruminants in Brazil. According to the infection area, the term enzootic stability is used when the transmission rate of *Babesia* spp. is sufficient to immunize the most susceptible calves before a loss of resistance, and to maintain enzootic stability, at least 75% of the herd must have been infected by the age of nine months (Smith, 1984; Tice et al., 1998).

The increased rate of infectious diseases is purportedly directly related to human-induced changes in land use, natural resource extraction, animal production systems, and wildlife trade (Karesh et al., 2012; Smith et al., 2017). The global climate impact is also an important factor to consider on tick-borne disease spread, specially related to temperature and moisture conditions, which can affect many of the physiological processes of ticks (Randolph, 2009). Despite the CNP being a CU, different activities are carried out in its territory, and subsistence farming is the primary source of income for the local population.

Although the situation regarding tick-borne disease is unknown, it is important to point out that the studied area presents suitable conditions for the emergence of outbreaks because the competent vector for the disease is present in the area. Furthermore, there are not many epidemiological data available related to hemoparasites in the region, as well as in small ruminants. This survey aimed to verify the occurrence of anti-*Babesia* spp. and anti-*A. marginale* antibodies using an enzyme-linked immunosorbent assay (ELISA) in domestic animals in the CNP, located in a semiarid region, and determined the risk factors for infection.

## Material and Methods

### Study area

**Figure 1:**
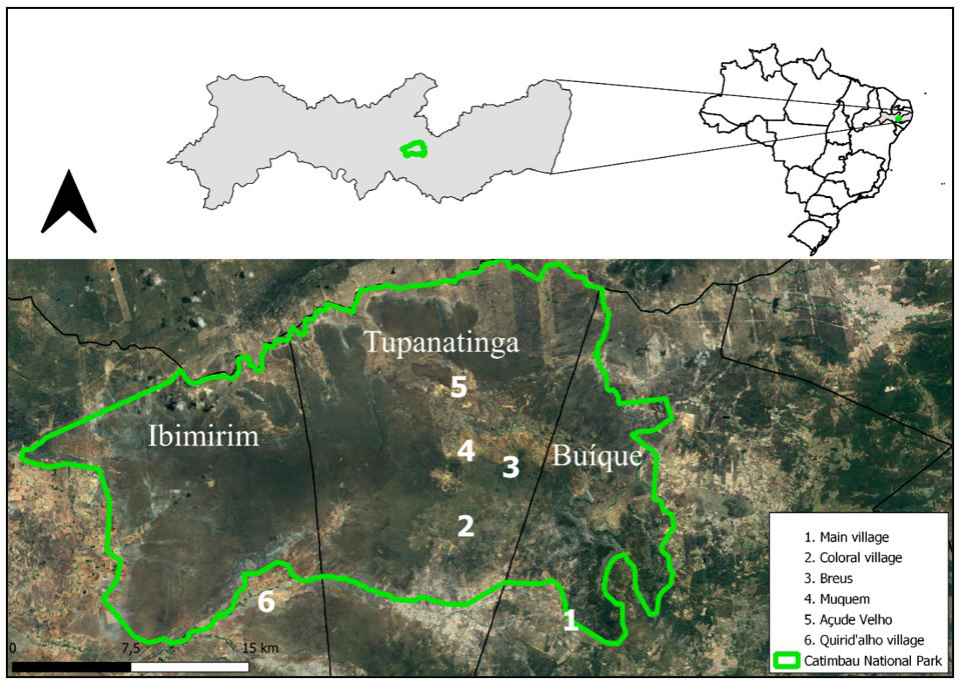
Collection sites of blood and ectoparasites of domestic animals in the geographic limits and surrounds of the CNP, representing the different regions studied.

The study was carried out at the CNP (8° 30' 57″ " S; 37° 20' 59"″ W) in Pernambuco State, which covers the municipalities of Buíque, Ibimirim, and Tupanatinga, covering an area of ​​62,294 ​​ha, with Caatinga as its biome, and Caatinga stricto sensu and Carrasco as phytophysiognomy (Rodal et al., 1998). Rainfall in the CNP is irregular, with annual values of 650 - 1,100 mm and an average annual temperature of 25ºC (Guimarães, 2002). The CNP, a CU, was created by decree law no. 4.340 of 08/22/2002, following federal law nº. 9.985 (SNUC Law) (Brasil, 2002), to preserve natural ecosystems of great ecological relevance and scenic beauty, enable scientific research, develop environmental education and interpretation activities, recreation in contact with nature, and ecological tourism (Santos, 2003). The study was conducted in six regions: Coloral Village, Açude Velho, Breus, Muquen, and Main Village in the Agrest region, and Quirid’alho Village located in Sertão (Figure 1). The selected sub-regions were chosen due to the being insert in the same National Park, although it showed differences regarding phytophysiognomy.

The selected areas have the following phytophysiognomy: Quirid’alho Village is characterized as Caatinga stricto sensu, in a region where indigenous families are concentrated, with small livestock, and is the only area in the Sertão region with a dry climate and low availability of water. Coloral Village (Caatinga stricto sensu) presents vegetation similar to that of Quirid’alho Village. The regions of Açude Velho, Muquen, and Breus are anthropized stricto sensu Caatinga, with large slopes, a dry climate, and many rocks, where firewood and grazing areas for small ruminants were explored.

### Domestic animals

Non-probability sampling (convenience sampling) was performed (Thrusfield & Christley, 2018); samples were collected in January, June, and October 2021, from 119 sheep, 119 goats, and 47 cattle inside the park and/or in its surroundings, according to the availability of livestock in the CNP. Different areas (Sertão and Agreste) were explored, using randomization as the criteria for animal sample. The team investigated the presence of ectoparasites and wounds and analyzed the general health.

### Blood collection

To collect blood from goats, sheep, and cattle, the animals were physically restrained aided by ropes carrying a halter and harness for cattle. Blood samples were obtained through venipuncture of the external jugular and/or coccygeal veins (cattle) and the external jugular veins (goats and sheep) using tubes containing EDTA. The samples were placed in tubes containing anticoagulants and stored in thermal boxes until further processing. To obtain the plasma, samples were centrifugated (3,000 g for 15 min), stored in 1.5 mL microtubes, identified, and stored at -20°C. In addition, a small amount of collected blood, approximately 0.5 mL per animal) was placed in 1.5 mL microtubes for DNA extraction.

### Collection and identification of ectoparasites

The animals were inspected using brushing and tweezers and all the ectoparasites found parasitizing the sampled animals were collected. After, the ectoparasites were properly stored in sealed plastic jars with lids containing small holes packed in thermal packs for later identification. In the laboratory, the specimens were kept for at least 72 h in a BOD incubator at 25°C and then identified according to Dantas-Torres et al. (2019).

### ELISA

MSP5 antigens (*A. marginale* recombinant protein) (Marana et al., 2009), BV60 antigens (*B. bovis* recombinant protein) (Suarez et al., 1991), currently known as RAP-1 (Suarez et al., 1993), and *B. bigemina* antigens (Machado et al., 1994) were used. Calves (five to six-month-old) free from hemoparasites, were splenectomized and inoculated intravenously with cryopreserved *B. bigemina* and were submitted to microscopic examination of Giemsa-stained blood smears daily, to identify the presence of parasites. Around the 8th and the 10th days after inoculation, the peak of parasitemia was observed (40 to 60%) and infected blood was collected in an equal volume of Alsever's solution. According to Martin et al. (1971), the blood was diluted (1:4) in saline solution and the infected erythrocytes were subjected to lyses with ammonium chloride. An ammonium chloride solution (0.8%) was prepared using pyrogen-free deionised water (Boyle, 1968), the solution was adjusted to the pH 7.4 at room temperature and then it was warmed to 37^o^C. Nine volumes of this solution were mixed with one volume of the suspension of infected erythrocytes. The mixture was incubated at 37^o^C for 3 min until hemolysis became evident. The mixture was centrifuged at 1,500 g for 10 min and the erythrocyte-free sediment was washed three times in sterile saline solution. The pellet was resuspended in 5 vols of PBS containing protease inhibitor (1 mM PMSF, 2 mM TPCK and 0.1 mM TLCK). *B. bigemina* free merozoites were disrupted by freezing/thawing cycles using liquid nitrogen. The supernatant obtained after centrifugation at 12,000 x g for 60 min at 4^o^C was stored at -20^o^C until required for ELISA (Machado, 1991). The samples were diluted to 1:200 for cattle and 1:100 for goats and sheep, and the plates were read after 45 min. To calculate the cutoff point (CP), the following formula was used: mean optical density of negative controls X 2.5 (constant–correction factor). Sera from animals known to be positive and negative (cattle, sheep, and goats, confirmed by immunofluorescence and PCR) were used as controls. The samples were classified based on the optical density value (O.D.), which was considered positive if the value was greater than or equal to the PC. The PCs used in cattle were: MSP5 = 0.247, BV60 = 0.295, and *B. bigemina* = 0.292; for goats, MSP5 = 0.208 BV60 = 0.251 *B. bigemina* = 0.228; and for sheep, MSP5 = 0.205, BV60 = 0.267, and *B. bigemina* = 0.231 (Machado et al., 1994, 1997; Ruiz et al., 2001; Juliano et al., 2007; Matos et al., 2017; Barbosa et al., 2021; Mushtaq et al., 2021).

### Epidemiological survey

A questionnaire was prepared and conducted among farmers, asking for technical information about the cattle, goats and sheep, such as, management, feeding, reproduction, free access to the park and vegetation, and contact with wild animals and ectoparasites in the area. All the domestic animals were physically examined by our team to determine their body score, dehydration level, presence of ectoparasites and skin wounds, to infer about their health status. This information was obtained to evaluate the risk factors for the transmission of these diseases and their circulation in the region, as well as the population’s knowledge regarding zoonoses. Information related to animal health, such as previous disease records, contact with wild and domestic animals, and current or previous ectoparasites, were collected for the risk factor analysis.

### Risk factor analyses

To analyze factors associated with positivity, categorical variables were subjected to univariable analysis, in which independent variables underwent an association analysis concerning the dependent variable (positivity in serological test/PCR). Variables with a P-value ≤ 0.2 in a chi-squared test were selected for multivariable analysis using robust Poisson regression with backward elimination. Continuous variables were included as covariates in the model. Correlation analysis was performed to check for collinearity between independent variables; for variables with strong collinearity (correlation coefficient > 0.9), one of the two variables was excluded from the multiple analyses based on biological plausibility (Dohoo et al., 2003). Confounding was assessed by monitoring the changes in the model parameters, and if substantial changes (i.e., > 20%) were observed in the regression coefficients, this was considered to indicate confounding. The interaction terms among the variables of the final models were analyzed by pairwise comparisons of estimated marginal means based on the original scale of the dependent variable using the Bonferroni test. The significance level adopted in the multivariate analysis was 5%, and the software used was SPSS version 21 for Windows (Software.informer, 2023).

The variables used in the risk analysis were breeding system (intensive, semi-intensive, or extensive), contact with wild animals (yes or no), abortion history (yes or no), free access to the park (yes or no), tick history (yes or no), flea history (yes or no), pale mucous membranes (yes or no), and mucous membranes (pale, normal, or ischemic). In addition, in this kind of study (cross-sectional), the association measures “odds ratio (OR)” and “relative risk (RR)” are not suitable according to Martinez et al. (2017) – reference below, so that we used the Prevalence Ratio (PR), an alternative for RR in cross-sectional surveys.

### Spatial analysis

The capture points of goats, sheep, and cattle, as well as their ectoparasites, were obtained with the aid of GPS for later elaboration of maps within each evaluated area, using georeferenced information systems that indicated the location of the captured specimens using the program QGis v. 2.18. 170.

## Results

### Samples

The distribution of samples was 84% (100/119) goats, 73.1% (87/119) sheep, and 60% (28/47) cattle inside the park, and 40% (19/47) cattle, 26.9% (32/119) sheep, and 16% (19/119) goats in the park surroundings. ELISA detected anti-*B. bovis* in 34% of cattle (16/47; titers μ= 0.28655, σ= 0.1285), 20.3% of goats (24/119; titers μ= 0.24759, σ=0.31754), and 16% of sheep (19/119; titers μ= 0.20555, σ=0.2251). For *B. bigemina*, antibodies were detected in 34% of cattle (16/47; titers μ= 0.28109, σ=0.12773), 15.2% of goats (18/119; tigers μ= 0.16159, σ=0.08672), and 9% of sheep (7/119; titers μ= 0.13253, σ=0.13214). Antibodies anti *A. marginale* were detected in 35.6% of goats (42/119; titers from μ= 0.2313, σ=0.21652), 34% of cattle (16/47; titers μ= 0.25309, σ= 0.12505), and 17% of sheep (20/119; titers μ= 0.15892, σ=0.14349) (Table 1). A total of 47 *Rhipicephalus microplu*s ticks were identified in cattle. There was not any goats or sheep infested at the time of the sample collection.

**Table 1:** Serological ELISA assays for diseases transmitted by ectoparasites (*Babesia* and *Anaplasma*), performed on ruminants, and collected at different points in the CNP and its surroundings.

Hosts	ELISA					
	*Babesia bovis*	Antibody titer	*Babesia bigemina*	Antibody titer	*Anaplasma marginale*	Antibody titer
Goat	20.3% (24/119)	0.264-1.125	15.2% (18/119)	0.229-0.504	35.6% (42/119)	0.208-1.390
Sheep	16% (19/119)	0.270-1.532	9% (7/119)	0.263-1.149	17% (20/119)	0.220-0.799
Cattle	34% (16/47)	0.318-0.579	34% (16/47)	0.337-0.570	34% (16/47)	0.251-0.621

### Serological assays

**Figure 2:**
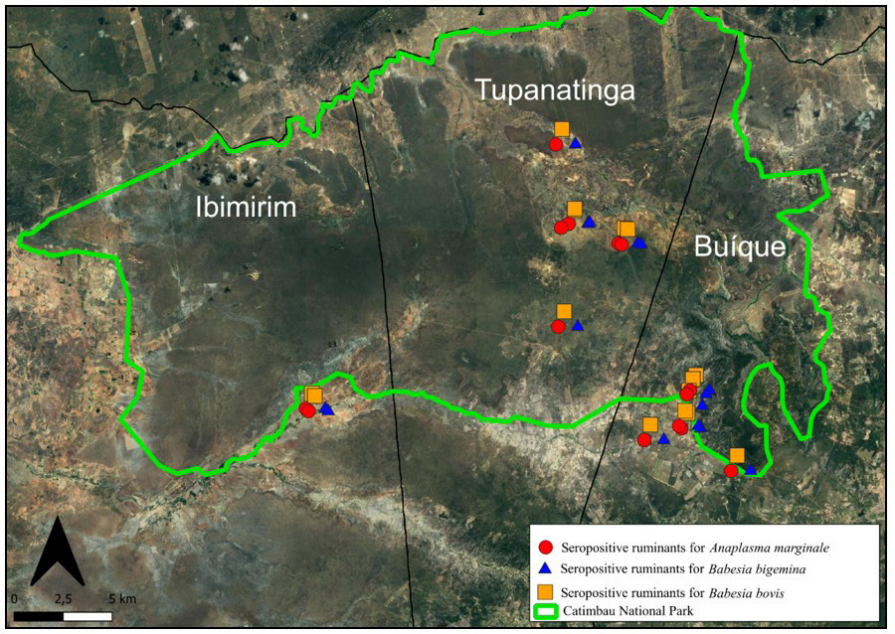
Geographical data of ruminants showing seropositivity in the ELISA test for multiple pathogens studied (*B. bovis*, *B. bigemina,* and *A. marginale*) in the CNP.

Regarding coinfection, the seropositive results were divided into the following four groups: *B. bovis* + *B. bigemina* (6.7% (1/15) cattle, 5.5% (1/18) sheep, and 3.6% (1/28) goats), ii. *B. bovis* + *A. marginale* (61.1% (11/18) sheep, 53.6% (15/28) goats, and 20% (3/15) cattle), and iii. *B. bigemina* + *A. marginale* (17.9% (5/28) of goats, 6.7% (1/15) of cattle), and iv. *B. bovis* + *B. bigemina* + *A. marginale* (66.7% (10/15) cattle, 27.8% (5/18) sheep, and 25% (7/28) goats (Table 2, Figure 2).

**Table 2:** Prevalence of coinfections in ruminants sampled from the CNP; they were seropositive for multiple agents using ELISA test (*Babesia bovis*, *Babesia bigemina*, and *Anaplasma marginale*).

Host	*B. bovis* + *B. bigemina*	*B. bovis* + *A. marginale*	*B. bigemina* + *A. marginale*	*B. bovis* + *B. bigemina* + *A. marginale*
Goat	3.6% (1/28)	53.6% (15/28)	17.9% (5/28)	25% (7/28)
Sheep	5.5% (1/18)	61.1% (11/18)	-	27.8% (5/18)
Cattle	6.7% (1/15)	20% (3/15)	6.7% (1/15)	66.7% (10/15)

A high prevalence of anti-*B. bovis* (54.2%), anti-*B. bigemina* (44.4%), and anti-*A. marginale* (38.1%) antibodies was found in Muquen. Higher seroprevalence was detected for *B. bovis* (42.1%), *B. bigemina (*57.1%), and *A. marginale* (57.1) in the Main Village area. For cattle, the area with the highest seroprevalence was also the Main Village, with 68.7% anti-*B. bovis,* 68.7% anti-*B. bigemina,* and 62.5% anti-*A. marginale* antibodies (Table 3).

**Table 3:** Geographical distribution of seropositive ruminants for *Babesia* and *Anaplasma* in ELISA collected in the CNP.

Collection site	*Babesia bovis*			*Babesia bigemina*			*Anaplasma marginale*		
	Goat	Sheep	Cattle	Goat	Sheep	Cattle	Goat	Sheep	Cattle
Quirid’alho Village	16.7% (4/24)	26.5% (5/19)	18.8% (3/16)	16.7% (3/18)	28.6% (2/7)	25% (4/16)	31% (13/42)	20% (4/20)	37.5% (6/16)
Breus	29.2% (7/24)	-	12.5% (2/16)	11.1% (2/18)	-	-	21.4% (9/42)	-	-
Muquen	54.2% (13/24)	31.6% (6/19)	-	44.8% (8/18)	12.3% (1/7)	6.3% (1/16)	38.1% (16/42)	35% (7/20)	-
Main Village	-	42.1% (8/19)	68.7% (11/16)	27.8% (5/18)	57.1% (4/7)	68.7% (11/16)	9.5% (4/42)	45% (9/20)	62.5% (10/16)

### Risk factors

The distribution of samples was based on convenience sampling. The risk factors for *B. bovis* in the serological assay showed that cattle from the Agreste region (Coloral Village, Açude Velho, Breus, Muquen, and Main Village) had a higher prevalence of antibodies when raised in a semi-intensive management system. Also, it was found a higher prevalence in older animals with a history of tick disease. The higher prevalence for *B. bigemina* was found in sheep with ischemic mucosa and those living inside the park. Older male cattle and sheep with ischemic mucosa presented a higher serological prevalence for *B. bigemina* as well. None of the independent variables was excluded because there was not strong collinearity. Risk factors for *A. marginale* were present only in goats, with a higher prevalence in animals from the Sertão region (Quirid’alho Village) (Table 4).

**Table 4:** Analysis of risk factors associated with exposure to the agents *B. bovis, B. bigemina,* and *A. marginale* in different species of domestic animals based on serological and molecular results.

Variable	Categories	Total number of animals	Number of positive animals (%)	Regression coefficient (β)	Prevalence ratio (PR)	95% CI (PR)	*P*-value
**Cattle - *Babesia bovis* (ELISA)**							
Region	Sertão	13	2 (15.4)	-	1	-	
	Agreste	30	14 (46.7)	0.469	1.59	1.06 - 2.38	0.024
Management system	Intensive	13	3 (23.1)	-	1	-	
	Semi-intensive	30	13 (43.3)	0.439	1.55	1.12 – 2.15	0.008
**Cattle - *Babesia bigemina* (ELISA)**							
Sex	Female	19	4 (21.1)	-	1	-	
	Male	24	12 (50)	0.391	1.48	1.10 – 1.99	0.010
Age	-	-	-	0.007	1.007	1.001 – 1.013	0.031
**Goat – *Babesia bovis* (ELISA)**							
Tick history	No	109	16 (14.7)	-	1		
	Yes	9	4 (44.4)	1.250	3.49	1.37 – 8.91	0.009
Age	-	-	-	0.020	1.020	1.001 – 1.39	0.040
**Goat – *Anaplama marginale* (ELISA)**							
Region	Agreste	99	28 (28.3)	-	1		
	Sertão	19	13 (68.4)	0.772	2.16	1.10 – 4.26	0.026
**Sheep – *Babesia bovis* (ELISA)**							
Oral mucosa	Normal/pale	117	18 (15.4)	-	1		
	Ischemic	1	1 (100)	0.525	1.69	1.05 – 2.73	0.032
Inside park	Yes	87	6 (6.9)	-	1		
	No	18	5 (27.8)	0.544	3.13	1.08 – 9.08	0.036
	Surroundings	13	8 (61.5)	0.595	4.96	1.54 – 15.91	0.007
**Sheep – *Babesia bigemina* (ELISA)**							
Oral mucosa	Normal/pale	117	7 (6)	-	1		
	Ischemic	1	1 (100)	1.525	4.59	1.68 – 12.56	0.003

### Questionnaire

In the questionnaire, 87.5% (7/8) of the goat farms had a semi-intensive system and 12.5% (1/8) had an extensive system; 100% (7/7) of the sheep farms had a semi-intensive system, and 50% (6/12) had a semi-intensive system and 50% (6/12) had an intensive system. In the intensive system, the animals were kept confined and provided food and water in the trough. In the semi-intensive system, the animals were left free during the day to feed inside the CNP in the Caatinga vegetation area and returned at the end of the day. In the extensive production system, animals were raised with free access to CNP. Furthermore, 87.5% (7/8) of goats, 85.7% (6/7) of sheep, and 50% (6/12) of cattle farms allowed animals access to the CNP.

Anemia history was reported in 42.8% (3/7) of sheep, 37.5% (3/8) of goats, and 33.3% (4/12) of cattle farms. Previous tick infestations were reported by owners in 50% (6/12) of cattle farms, 14.3% (1/7) of sheep farms, and 12.5% (1/8) of goat farms. No farm manager reported any environmental treatments to control ectoparasites. Of the total properties, 30.4% (7/23) did not have a vaccination protocol for any disease, and 13% (3/23) did not have a vaccination routine, including two goat farms and one cattle farm (Table 5).

**Table 5:** Production system and health data obtained from a questionnaire referring to the ruminants sampled in the CNP.

Questions	Species		
	Goat	Sheep	Cattle
Management system			
Intensive	-	-	6 (26.08%)
Semi-intensive	7 (30.43%)	7 (30.43%)	6 (26.08%)
Extensive	1 (4.35%)	-	-
Park access	7 (30.43%)	7 (30.43%)	6 (26.08%)
Sanitary status			
Specialized assistance	0	0	0
Disease history	2 (8.69%)	2 (8.69%)	4 (17.39%)
Ticks	1 (4.35%)	1 (4.35%)	6 (26.08%)
Ectoparasites control	0	0	0
Vaccination	84 (70.58%)	119 (100%)	44 (93.61%)

## Discussion

The seroprevalence for the studied agents was verified in all properties; however, its prevalence was lower than that in other regions of Brazil. The highest percentages were found in goats and sheep for *A. marginale*, and cattle showed the same prevalence for all three agents. *B. bigemina* had the lowest prevalence in goats and sheep compared with the prevalence of the other two agents. The higher prevalence of *A. marginale* might have been related to mechanical transmission by fly bites, which play an important role in the epidemiology of anaplasmosis in areas where environmental conditions do not favor the vector perennially (Costa et al., 2013).

Santos et al. (2017) evaluated the seroprevalence of *A. marginale*, *B. bigemina*, and *B. bovis* in cattle in the municipality of Petrolina using an IFAT assay, revealing prevalence rates of 35.0%, 35.9%, and 32.3%, respectively. Also, in Ouricuri, the seroprevalence was 45.5%, 38.6%, and 54.9%, respectively, showing results similar to those found in Petrolina; both regions are located in semiarid parts of the Northeast, with Caatinga and the CNP being the parts with the most abundant vegetation among these regions. This study was conducted in the same state and region as in the present study, which provided data that served as the basis of the present study’s analyses. Costa et al. (2013) reported that the prevalence of *A. marginale*, *B. bovis,* or *B. bigemina* infection was generally low (< 50%) or sometimes zero among adult cattle raised in the Caatinga biome.

Barros et al. (2005) conducted a study in the state of Bahia, in the Sertão region, where different enzootic areas were found within the same state, having stable enzootic areas reported in Senhor do Bonfim (86% *B. bovis* and 90.8% *B. bigemina*) and Euclides da Cunha (95.5% *B. bovis* and 91.3% *B. bigemina)*. In contrast, Uauá (63.7% *B. bovis* and 53% *B. bigemina*) and Juazeiro (56.4% *B. bovis* and 54.8% *B. bigemina*) were characterized as enzootic unstable areas. Even among cities within the same state, differences in rainfall and relative humidity can directly influence the survival of a vector. Temperature and humidity are important factors related to the development of *Rhipicephalus microplus*, and humidity determines the different epidemiological conditions found in the semiarid region of Bahia (Barros et al., 2005).

In a study in the Caatinga biome, the average temperatures were generally constant, approximately 30–35 °C, regardless of the time of year, indicating that this factor does not play an important role in the seasonal variations of *R. microplus*. Conversely, rainfall in this region is highly variable, which may be a determinant in the life cycle of *R. microplus* in the Caatinga (Barros et al., 2017). It is important to explore the tick fauna in the region because these ticks are direct related to the spread of a number of pathogens to animals and humans (Nava et al., 2017).

Areas of enzootic instability are marked by a prevalence of infection between 20% and 75% in animals older than nine months (Mahoney & Ross, 1972). The concept of enzootic stability–instability used in babesiosis can also be applied to anaplasmosis (Guglielmone, 1995). Potential outbreaks of babesiosis and anaplasmosis can occur in cattle from humid areas, where the prevalence of ticks is higher in herds from the semiarid region during the rainy season or vice versa (Barros et al., 2005). Although *R. microplus* exhibits poor survival in the dry season, when cattle from areas with ticks are introduced into the herd at the beginning of the rainy season, the ectoparasite exhibits better survival and multiplication (Costa-Júnior et al., 2009), which can cause outbreaks.

The small ruminants sampled did not exhibit parasitism by ticks, blood-sucking flies, or tabanids at the time of collection. Costa et al. (2011) conducted a study in the Sertão region of Paraíba state, a region with similar climatic conditions as that of Caatinga, reporting that clinical cases of CTF were more frequently caused by anaplasmosis than by babesiosis, which might have been associated with the forms of transmission. *R. microplus* is the main biological vector of *Babesia* and is widely distributed in tropical and subtropical regions (Guglielmone et al., 1990). The greater variety of vectors that transmit *A. marginale* may explain the variation in the prevalence rates detected between the agents, such as the involvement of blood-sucking flies and tabanids in transmission, in addition to the transmitting tick, which may increase in prevalence in herds (Castañeda-Ortiz et al., 2015).

Tabanids can mechanically or biologically transmit various viruses, such as equine infectious anemia virus, bovine leukosis virus, protozoa, and bacteria, such as *A. marginale* (Foil, 1989; Foil and Hogsette, 1994). Biting flies, specifically deer and horse flies (Diptera: Tabanidae), can serve as mechanical vectors for *A. marginale* in cattle (Baldacchino et al., 2014). This transmission occurs when insects are interrupted during blood feeding on an infected host, and when they feed on another host, they immediately transfer the agent to a susceptible host (Desquesnes et al., 2009). Semiarid humidity is not favorable for the development of tabanids, except during the short rainy season. The epidemiological factor affecting its high prevalence in this region may be the persistence of infection by *A. marginale* acting as a reservoir (Barros et al., 2005).

Costa et al. (2018) theorized that the transmission of anaplasmosis in the semiarid region of Paraíba occurs mainly by tabanids and wild animals, such as the gray brocket deer (*Mazama gouazoubira*), which may be important reservoirs of this agent (Costa et al., 2018). This species has already been described in the CNP and is found more often in forested landscape areas (Alves et al., 2020), and may have an important role as a reservoir in the park.

Costa et al. (2011) registered 24 outbreaks of CTF in the Sertão region of Paraíba, 18 of which were caused by anaplasmosis, two by *B. bigemina*, two by *Babesia* spp., and two by a coinfection between *A. marginale* and *Babesia* spp., presenting a high lethality, being classified as an area of enzootic instability, as well as the CNP. Of the 24 outbreaks in Paraíba, 22 occurred at the end of the rainy season and the beginning of the dry season, which is the period that facilitates greater survival of vectors, given the greater humidity as well as the acquisition of new animals for the herd.

Considering the concept of enzootic stability used by Mahoney & Ross (1972), it is possible to infer that these epidemiological data in regions with characteristics similar to those of the study site suggest the possibility of outbreaks in the CNP. To classify the area as with enzootic stability, it would be necessary to analyze different criteria, as the generation of data relating to infection rates of ticks by pathogens, rates of tick infestation of cattle, serial seroprevalence in cattle, and disease incidence in animals of various genotypes (Jonsson et al., 2012). Some intervention measures to ameliorate the outbreaks of CTF and tick population would be the use of vaccines, synthetic and botanical acaricides, producer education and the monitoring and management of drug resistance as well as of tick and host populations (de la Fuente et al., 2015).

Coinfection is more commonly observed in endemic areas when cattle are parasitized by vectors infected by multiple pathogens or different vectors transmitting multiple pathogens (Diuk-Wasser et al., 2016). Evidence suggests the possibility of coinfection, showing that the simultaneous presence of the three parasites in animals and other environmental factors, such as air humidity, temperature, and rain, may interfere with this event (Furlong et al., 2002; Santos et al., 2017). Understanding and identifying coinfection is important due to several pathogens influencing pathogenesis in the host and hindering diagnoses (Hakimi et al., 2019). A greater number of goats and sheep were infected by the two agents, whereas cattle exhibited the highest prevalence for the three agents.

Ferreira et al. (2022) reported that the most diverse coinfections were observed between *B. bovis* and *B. bigemina*, *B. bovis* + *A. marginale*, *B. bigemina* + *A. marginale*, and *B. bovis* + *B. bigemina* + *A. marginale*. In the Northeast, the prevalence of coinfection in cattle was approximately 4%, with the interaction of the three agents. Obregón et al. (2019) found a tendency toward coinfection with *B. bovis* and *A. marginale* in calves, as well as infection by *B. bigemina* in young animals. Santos et al. (2017) assessed cattle in the state of Pernambuco, Caatinga biome, using the same serological tool used in this study, identifying coinfection with *Anaplasma* spp. and *Babesia* spp. in 32.1% and 31.6% of animals from Ouricuri and Petrolina, respectively.

The interpretation of serological results should be performed with attention. A positive result would indicate exposure to parasite infection, but does not accurately shows what species are infecting the animal. In addition, a positive serological result can also demonstrate either past or current infection. Also, the cross-reaction phenomenon is a common problem among the different *Babesia* species (Alvarez et al., 2019).

As for the information obtained during the study, in the semi-intensive breeding system, producers usually keep their animals sheltered at night and leave them free to feed on the Caatinga vegetation, feeding on the natural pasture of the CNP and having contact with free-ranging wild animals and other domestic animals in the area. In the extensive breeding system, the animals are free in Caatinga. In Brazil, management systems and climatic conditions directly interfere with the vector cycle (Santos et al., 2001; Ríos-Tobón et al., 2014).

The anemia found in these animals might have been related to other factors, as gastrointestinal and external parasitism are common causes of anemia in small ruminants (Johns & Heller, 2021). Among the symptoms that the different species of *Babesia* can cause, there is anemia and severe thrombocytopenia (Irwin, 2009), which led us to consider this clinical sign as a possible risk factor, being evaluated by our team through ocular conjunctiva. Infections by *B. bovis* and *A. marginale* are also related to late abortions, stillbirths and neonatal deaths in cattle (Henker et al. 2020). With this is mind, reproductive history was also considered in this work as a potential factor of risk.

A higher prevalence of *B. bovis* was observed in the Agreste region for cattle raised in a semi-intensive management system and for *A. marginale* in goats from the same region. The vegetation and climatic conditions of the Agreste region can influence the prevalence of these agents, thereby improving the survival conditions of the vectors.

In goats, a higher prevalence was found in older animals with a history of ticks. In the present study, no extensive livestock was reported, and only intensive and semi-intensive systems for cattle were reported.

Sheep also presented as a risk factor for *B. bovis* the presence of hyperemic mucosa, as well as being within the limits of the park. A higher prevalence of *B. bigemina* was observed in older male cattle and sheep with hyperemic mucosa. Although no ticks were found parasitizing goats at the time of collection, ticks had been reported in some of the farms in which some animals were found, which could parasitize these animals at some point. Risk factors for *A. marginale* were present only in goats, with a higher prevalence in animals from the Sertão region.

Small herds represent a large part of the local economy, as many people in the region support their families by raising animals for family-employed farmers. Knowledge about these diseases and how they can influence animal health and, consequently, bring losses to this economic modality is crucial for local producers and the local population, as it is important to collect epidemiological information in regions of epidemiological silence.

In conclusion, this work found *Babesia bigemina, Babesia bovis* and *Anaplasma marginale* antibodies in ruminants in the Catimbau National Park region. This was the first report, in an area of epidemiological silence, on the presence of anti-*B. bigemina*, anti-*B. bovis*, and anti-*A. marginale* antibodies in ruminants from the CNP in the Caatinga biome. These results provide a warning to rural producers, as these agents can lead to significant economic losses that directly affect the local economy because they are mostly composed of agricultural production and small farms. This study demonstrated that the incidence of CTF occurred heterogeneously in the region, confirming the status of the enzootic instability area for CTF. The Caatinga biome has great relevance in Brazil, as it presents a rich fauna and flora, and the results of the present study can be used as a reference for future studies in the area. Limited information is available about these agents in semiarid regions, so this work provides relevant data.
